# Flavonoids, Antioxidant Potential, and Acetylcholinesterase Inhibition Activity of the Extracts from the Gametophyte and Archegoniophore of *Marchantia polymorpha* L.

**DOI:** 10.3390/molecules21030360

**Published:** 2016-03-17

**Authors:** Xin Wang, Jianguo Cao, Yuhuan Wu, Quanxi Wang, Jianbo Xiao

**Affiliations:** 1 Insititute of Applied Ecology, Chinese Academy of Sciences, Shenyang 110016, China; xinwang2015@yahoo.com; 2 University of Chinese Academy of Sciences, 19A Yuquan Road, Beijing 100049, China; 3 College of Life and Environmental Sciences, Shanghai Normal University, Shanghai 200234, China; cao101@shnu.edu.cn; 4 College of Life and Environmental Sciences, Hangzhou Normal University, Hangzhou 310036, China; 5 Shanghai Key Laboratory of Plant Functional Genomics and Resources, Chinese Academy of Sciences, Shanghai Chenshan Botanical Garden, Shanghai 200234, China; 6 State Key Laboratory of Quality Research in Chinese Medicine, Institute of Chinese Medical Sciences, University of Macau, Taipa, Macau; jianboxiao@yahoo.com

**Keywords:** *Marchantia polymorpha* L*.*, flavonoids, antioxidant potential, archegoniophore, gametophyte, acetylcholinesterase inhibition

## Abstract

*Marchantia polymorpha* L. is a representative bryophyte used as a traditional Chinese medicinal herb for scald and pneumonia. The phytochemicals in *M. polymorpha* L. are terpenoids and flavonoids, among which especially the flavonoids show significant human health benefits. Many researches on the gametophyte of *M. polymorpha* L*.* have been reported. However, as the reproductive organ of *M. polymorpha* L*.*, the bioactivity and flavonoids profile of the archegoniophore have not been reported, so in this work the flavonoid profiles, antioxidant and acetylcholinesterase inhibition activities of the extracts from the archegoniophore and gametophyte of *M. polymorpha* L*.* were compared by radical scavenging assay methods (DPPH, ABTS, O^2−^), reducing power assay, acetylcholinesterase inhibition assay and LC-MS analysis. The results showed that the total flavonoids content in the archegoniophore was about 10-time higher than that of the gametophyte. Differences between the archegoniophore and gametophyte of *M. polymorpha* L. were observed by LC-MS analysis. The archegoniophore extracts showed stronger bio-activities than those of the gametophyte. The archegoniophore extract showed a significant acetylcholinesterase inhibition, while the gametophyte extract hardly inhibited it.

## 1. Introduction

Bryophytes, as the oldest known land plants, are of great significance in phylogenetic evolution. Many bryophyte plants are traditionally used to treating illnesses of the cardiovascular system, bronchitis, and burns and also possess antimicrobial, anticancer, antifungal, antimicrobial activity [1,2,3]. Approximately, 23,000 bryophyte species exist in the world, among which some 3021 species are found in China and about 50 species are used in medicine [4]. According to the analysis and statistics on the literature about the flavonoids of bryophytes, the species with reported flavonoid data only account for about 1.4% of the total number of bryophytes found in China.

Flavonoids, as plant secondary metabolites with over 10,000 known structures [5,6], have vital functions in plant growth and development [7]. Evidence based on epidemiological and pharmacological data has shown that the flavonoids play an important role in preventing and managing modern diseases such as cancer [8]; diabetes [5,9]; HIV [10]; inflammation and obesity [11]. At present, research on flavonoids almost all focus on spermatophytes, but seldom on bryophytes.

*Marchantia polymorpha* is a representative bryophyte and the gametophyte of *M. polymorpha* L*.* is traditionally used to cure cuts, fractures, poisonous snake bites, burns, scalds, and open wounds [4]. Many studies on the gametophyte of *M. polymorpha* L*.* have been reported. The extracts of *M. polymorpha* L. exhibited antifungal activity, antibacterial and antioxidant activities [12,13,14]. The main bioactive compounds in *M. polymorpha* L. are terpenoids, bis[bibenzyls] and polyphenols, especially flavonoids [15,16]. However, the bioactivity and flavonoids profiles of different parts of *M. polymorpha* L*.* have not been reported. Herein, the flavonoid profiles, antioxidant potential, and acetylcholinesterase inhibition activity of the extracts from the gametophyte and archegoniophore of *M. polymorpha* L*.* were compared.

## 2. Results and Discussion

### 2.1. Total Flavonoid Contents in M. polymorpha L.

**Figure 1:**
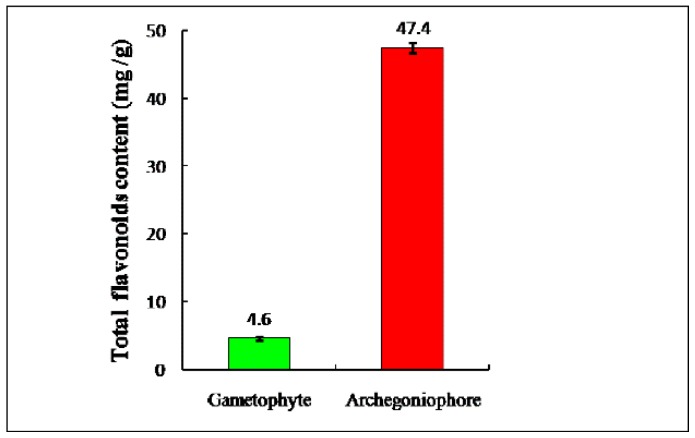
Total flavonoids contents of gametophyte and archegoniophore of *M. polymorpha* L*.* (*n* = 3).

The total flavonoids contents in the gametophyte and archegoniophore of *M. polymorpha* L*.* were determined as 4.62 ± 0.24 mg/g and 47.42 ± 0.76 mg/g, respectively (Figure 1), showing that the otal flavonoids content of the archegoniophore was ten times as haigh as that of the gametophyte.

The total flavonoids contents of 132 samples of bryophytes have also been determined, which ranged from 1.0 mg/g to 5.0 mg/g. It was found that the flavonoids content in the archegoniophore of *M. polymorpha* L*.* was also the highest in all the bryophyte parts. As secondary metabolites, flavonoids were generally regarded to be associated with growth. Some experts had noticed that quantitative variations in flavonoids when the plant moved into its reproductive phase [17], and then the vital functions of flavonoids on reproductive organs were reported [18,19,20]. As the female reproductive organ of *M. polymorpha* L*.*, the archegoniophore also showed quantitative flavonoid variations, which is consistent with the abovementioned view.

### 2.2. Antioxidant Activity

**Figure 2:**
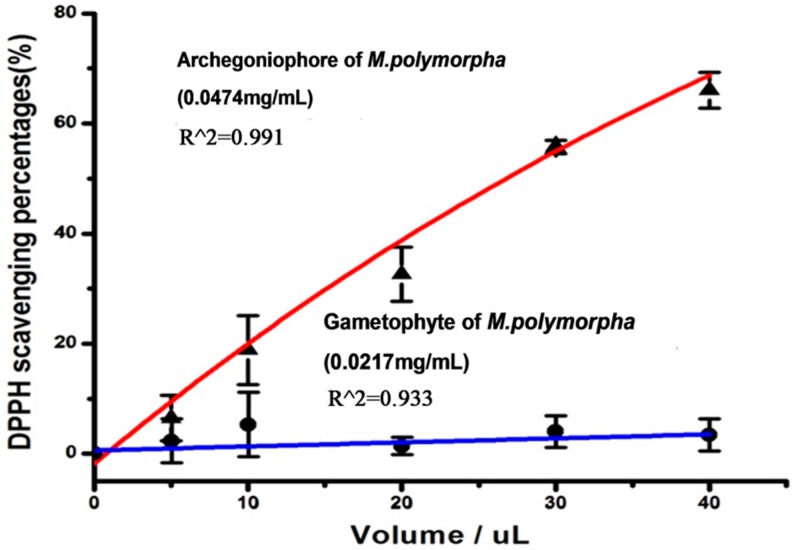
The DPPH free radical scavenging potential of the gametophyte and archegoniophore of *M. polymorpha* L. (*n* = 3).

The DPPH free radical scavenging activities of the extracts from the archegoniophore are shown in Figure 2. With increasing doses from 5.0 to 40.0 μL, the DPPH free radical scavenging potential observably increased. Forty μL of the extract from the archegoniophore could scavenge about 68% of the free radicals. However, the DPPH free radical scavenging potential of the extracts from the gametophyte of *M. polymorpha* L. was far lower than that of the archegoniophore. The DPPH·radicals scavenging activity IC_50_ of the archegoniophore extract was determined as 1.3 μg/mL.

**Figure 3:**
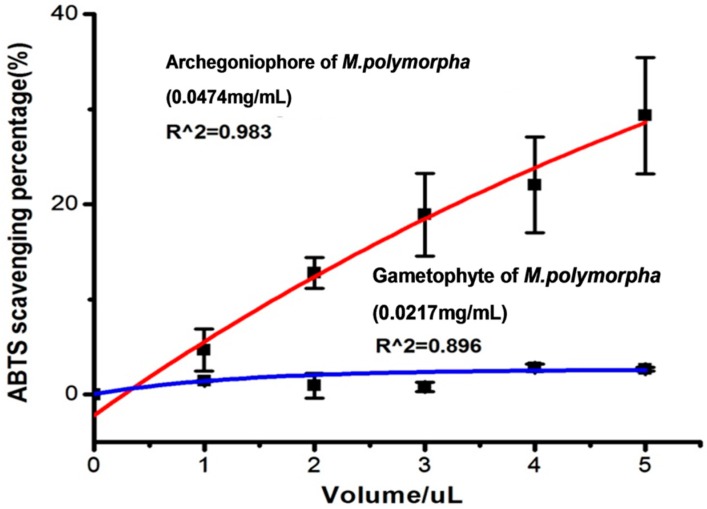
The ABTS radical scavenging potential of flavonoids extract from gametophyte and archegoniophore of *M. polymorpha* L*.* (*n* = 3).

The ABTS·radical scavenging potential of the extracts from the archegoniophore of *M. polymorpha* L*.* increased with increasing volume from 1.0 to 5.0 μL (Figure 3). Furthermore, the gametophyte extracts of *M. polymorpha* L. showed a lower scavenging ability than that of the archegoniophore. The IC_50_ of the archegoniophore extract was 3.0 μg/mL.

**Figure 4:**
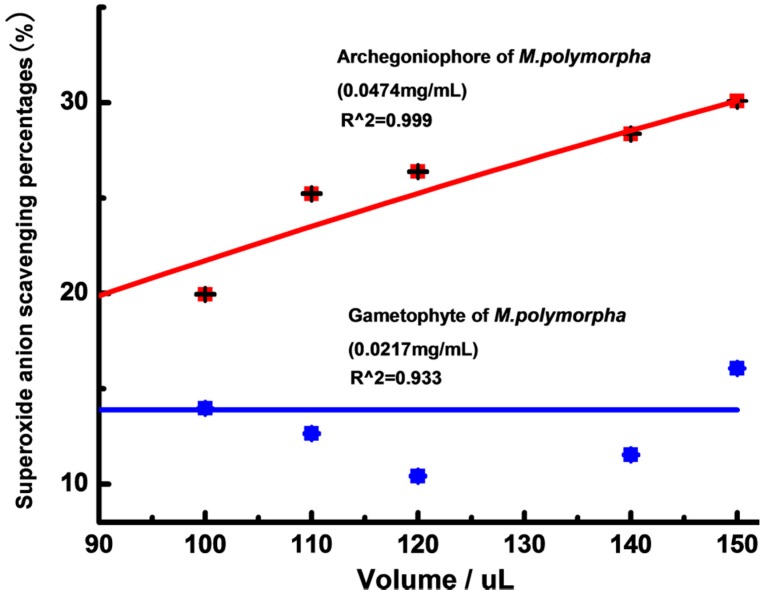
The superoxide anion scavenging potential of gametophyte and archegoniophore of *M. polymorpha* L*.* (*n* = 3).

The superoxide anion scavenging activity of the extracts from the archegoniophore and gametophyte of *M. polymorpha* L. is shown in Figure 4. With the increasing volumes from 100 to 150 μL, the superoxide anion scavenging potential of the extracts from the archegoniophore increased slightly, while the extracts from the gametophyte of *M. polymorpha* L. hardly showed any superoxide anion scavenging activity.

**Figure 5:**
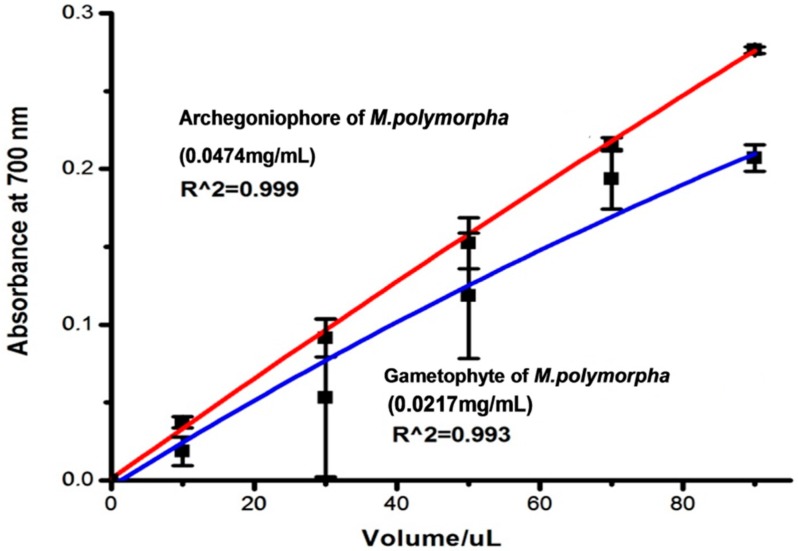
The reducing power of archegoniophore and gametophyte of *M. polymorpha* L*.* (*n* = 3).

Reducing powers of the extracts from the archegoniophore and gametophyte were tested, and the results are shown in Figure 5. Within the range of 20–160 μL of extract, the extracts from the archegoniophore exhibited higher activity than that of gametophyte.

**Figure 6:**
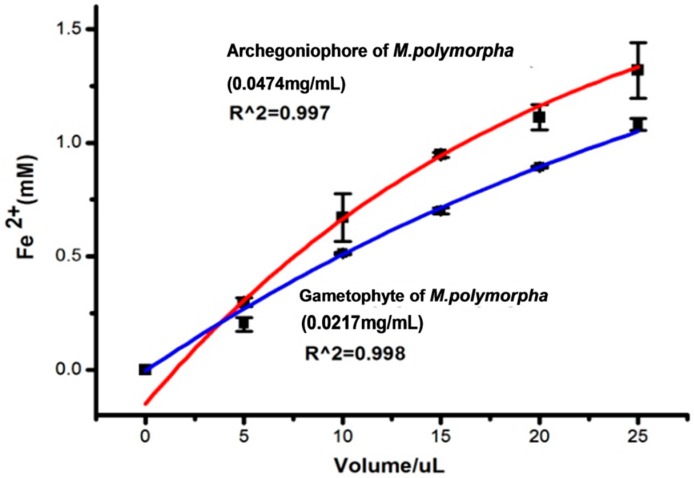
Antioxidant power by FRAP assay of archegoniophore and gametophyte of *M. polymorpha* (*n* = 3).

The FRAP assay was used to measure the antioxidant and reductive capacity of the extracts from the archegoniophore and gametophyte of *M. polymorpha* L*.* (Figure 6). The results illustrated that the extracts from the archegoniophore and the gametophyte of *M. polymorpha* L*.* both possessed antioxidant and reductive activity. With the increasing volume, the antioxidant and reductive capacity improved. Within the range of 5 to 25 μL, the activity of the extracts from the archegoniophore was stronger than that of the gametophyte.

In summary, the extracts from the archegoniophore showed more efficient scavenging activity on DPPH radical, ABTS radical and superoxide anion than those of the gametophyte. In the Fe^2+^ reducing power assay, minor differences between the archegoniophore and gametophyte were found, which suggested that the levels of archegoniophore and gametophyte constituents capable of reducing Fe^2+^ were almost same.

**Figure 7:**
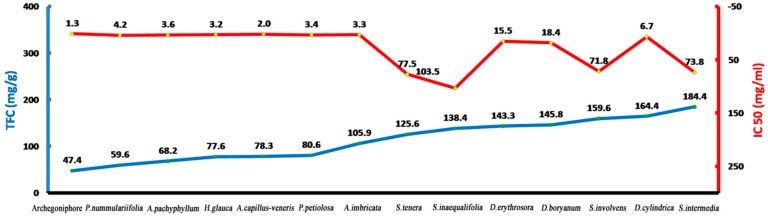
The comparison of total flavonoids content (TFC) and IC_50_.

The extracts with higher concentrations of total flavonoids usually showed stronger DPPH radical scavenging activity [21]. Here, the extracts from the archegoniophore showed higher total flavonoid content and stronger free radical scavenging activity. Although the archegoniophore results support the above viewpoint, the total flavonoids concentrations and DPPH radical scavenging activity IC_50_ values from this work were compared with those reported for other fern species, the orders from left to right were archegoniophore, *Pyrrosia nummulariifolia*, *Athyrium pachyphyllum*, *Hicriopteris glauca*, *Adiantum capillus-veneris*, *Pyrrosia petiolosa*, *Araiostegia imbricata*, *Selaginella tenera*, *Selaginella inaequalifolia*, *Dryopteris erythrosora*, *Dryoathyrium boryanum*, *Selaginella involvens*, *Selaginella intermedia* (Figure 7) [22,23,24]. The higher flavonoids content did not always show a positive correlation with the antioxidant activity.

### 2.3. AChE Inhibition Activity

**Figure 8:**
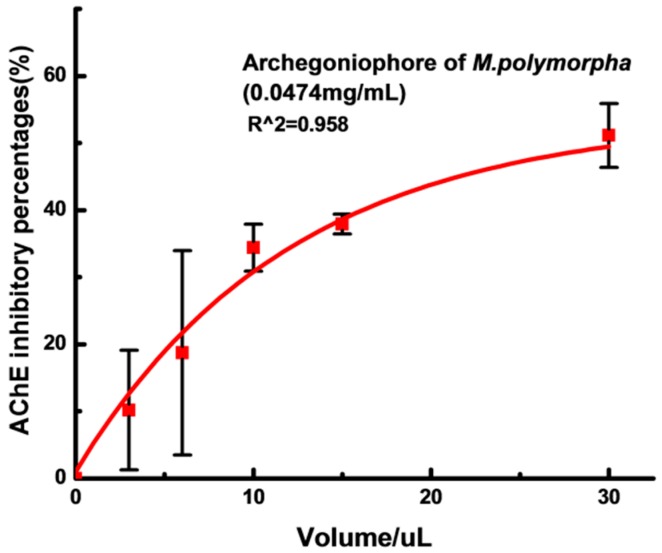
AChE inhibitory activity of archegoniophore of *M. polymorpha* L*.* (*n* = 3).

AChE inhibitors are chemicals that inhibit AChE for—Alzheimer’s disease (AD) [25]. AChE inhibitors were used first to treat glaucoma, but nowadays AChE has proven to be the most viable therapeutic target for symptomatic improvement in AD [26]. As shown in Figure 8, the extracts from the archegoniophore exhibited a dose-dependent inhibition against AChE (IC_50_ = 0.1256 mg/mL). However, the gametophyte extracts hardly inhibited the AChE activity.

### 2.4. Identification of Flavonoids in Extracts from the Archegoniophore and Gametophyte of M. polymorpha L.

**Figure 9:**
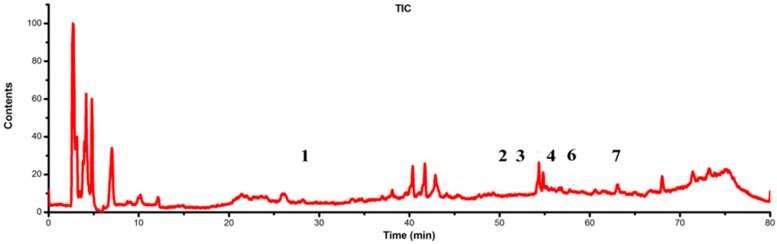
ESI-MS chromatograms of flavonoid extract of *M. polymorpha* L. gametophyte*.*

**Figure 10:**
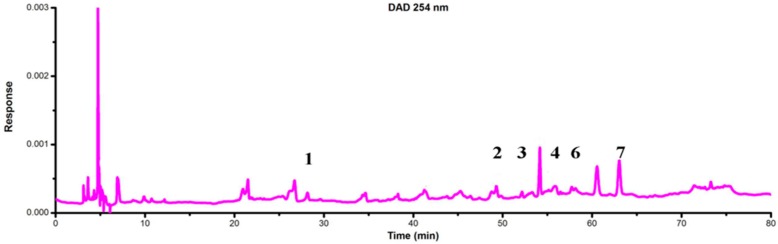
DAD (254 nm) chromatograms of flavonoid extract from *M. polymorpha* L. gametophyte*.*

**Figure 11:**
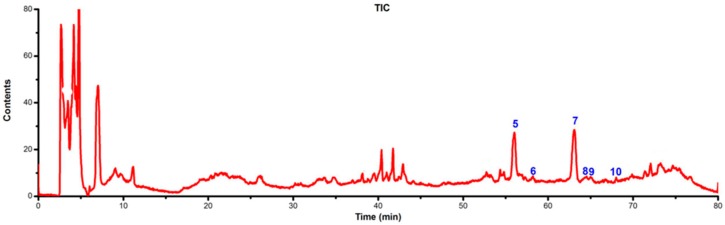
ESI-MS chromatograms of flavonoid extract of *M. polymorpha* L. archegoniophore.

**Figure 12:**
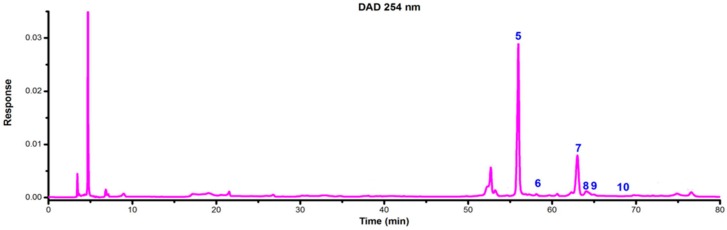
DAD (254 nm) chromatograms of flavonoid extract from *M. polymorpha* L. archegoniophore.

LC-DAD-ESI/MS data were used to identify the flavonoids. The retention time (t_R_), UV_λmax_ value, the molecular ions and structures of the flavonoids are listed in Table 1. TIC chromatograms and DAD (254 nm) chromatograms of the extracts from the gametophyte and archegoniophore of *M. polymorpha* L*.* are shown in Figure 9, Figure 10, Figure 11 and Figure 12 respectively.

From the comparison with chromatograms, mass spectral and UV_λmax_ reported data, it was tentatively identified that there were 10 flavonoids in the archegoniophore and gametophyte extracts (Table 1).

With UV_λmax_ at 292, and 344 nm, and molecular ions at *m*/*z* 595.1 [M + H]^+^, 593.2 [M − H]^−^, peak 1 was tentatively identified as kaempferol-3-*O*-rutinoside. Peak 2 with UV_λmax_ at 268, 300 and 336 nm, and molecular ions at *m*/*z* 609.2 [M + H]^+^, 607.1 [M − H]^−^, was tentatively identified as chrysoeriol 7-*O*-neohesperidoside [27]. For peak 3, the positive ESI-MS spectrum gave a molecular ion at *m*/*z* 433.1 [M + H]^+^, whereas the negative ESI-MS spectrum at *m*/*z* 431 [M − H]^−^, and the UV spectrum showed characteristic flavone absorptions at 268, 288 and 340 nm, so it was tentatively identified as apigenin 7-*O*-glucoside [28]. Peak 4 had UV_λmax_ at 258 and 330 nm, like baicalein 6,7-di-*O*-β-gluco-pyranuronoside and molecular ions at *m/z* 623.1 [M + H]^+^, 621.1 [M − H]^−^ [29]. Peak 5 had UV_λmax_ at 254 and 348 nm, which was similar to kaempferol, and molecular ions at *m*/*z* 287 [M + H]^+^, 285 [M − H]^−^, 571 [2M − H]− [30]. For peak 6, the positive ESI-MS spectrum gave a molecular ion at *m*/*z* 447.1 [M + H]^+^, whereas the negative ESI-MS spectrum peak at *m*/*z* 445 [M − H]^−^, and the UV spectrum which showed characteristic flavone absorptions at 268 and 336 nm, allowed this product to be tentatively identified as apigenin 7-*O*-glucuronide [31]. The molecular ions of peak 7 were at *m*/*z* 271 [M + H]^+^ in the positive ESI-MS spectrum and 269 [M − H]^−^ in the negative ESI-MS spectrum. This, together with the UV_λmax_ at 268 and 338 nm tentatively identified it as apigenin [31]. For peak 8, the UV_λmax_ at 266 and 340 nm, and molecular ions at *m*/*z* 301.1 [M + H]^+^, and 299 [M − H]^−^, tentatively identified it as chrysoeriol [31]. Peak 9 and peak 10 had similar retention times (t_R_), but the UV_λmax_ of peak 9 were at 254 and 348 nm with *m*/*z* at 463 [M + H]^+^, 461 [M − H]^−^, and the UV_λmax_ of peak 10 were at 266 and 366 nm with *m*/*z* at 639.1 [M + H]^+^, 637.1 [M − H]^−^, so they were tentatively identified as luteolin 3′-*O*-β-d-glucuronide [30], and tricin-7-*O*-rutinoside [32], respectively. 

Apigenin-7-*O*-β-D-glucuronide and apigenin had been previously reported in the gametophytes of *M. polymorpha* L*.* [33], and now apigenin 7-*O*-glucuronide and apigenin were also found in the archegoniophore. It was thus clear that there are no consistent differences in the flavonoid patterns of gametophytes and archegoniophore of *M. polymorpha* L*.*

## 3. Materials and Methods

### 3.1. Plant Materials

**Figure 13:**
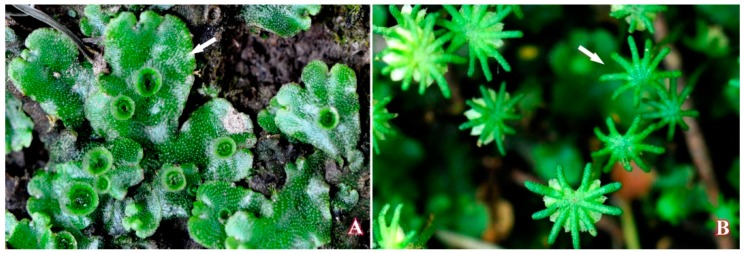
Gametophyte (**A**) and archegoniophore (**B**) of *M. polymorpha* L*.*

Gametophytes and archegoniophores of *M. polymorpha* L*.* (Figure 13) were collected from Mountain Tianmu National Natural Reserve, Zhejiang Province, China. The plant was identified by Prof. Yuhuan Wu. Voucher specimens (Gametophyte-2013070447; Archegoniephore-2013042116) are kept in the HTC of the College of Life & Environmental Science, Hangzhou Normal University.

### 3.2. Chemicals and Reagents

Rutin (purity > 99.0%), 2,2-diphenyl-1-picrylhydrazyl (DPPH), 2,2′-azinobis-(3-ethylbenzothiazoline-6-sulfonic acid) (ABTS), 2,4,6-tri-2-pyridyl-s-triazine (TPTZ), acetylthiocholine (ATCh), and AChE were purchased from Sigma Co. (Shanghai, China). Nitrotetrazolium blue chloride (NBT), phenazine methosulfate (PMS), nicotinamide adenine dinucleotide (NADH) and 5, 5′-dithiobis-(2-nitrobenzoic acid) (DTNB) were purchased from Aladdin Reagent Int. (Shanghai, China). Other reagents were of analytical grade, except for acetonitrile, which was HPLC grade and purchased from Thermo Fisher Scientific (Shanghai, China).

### 3.3. Preparation of Plant Extracts

Fresh plant materials, after being cleaned and dried under shady conditions, were dried at 75 °C for 48 h, and then powdered and filtered through a 40-mesh screen. The dried samples (1.00 g) were separately extracted with 60% ethanol (25 mL) for 2 h at 50 °C. Then, ultrasound-assisted extraction was performed for 20 min, the extraction processes were repeated twice. Finally, the mixture was filtered via a vacuum suction filter pump, and the extract solutions, which would be used to measure the total flavonoids content, were collected. Eight mL of extract solution was extracted twice with petroleum ether for removing the chlorophyll, and the residue solution was concentrated to dryness by evaporation on a rotary evaporator, and then dissolved with ethanol. Before testing the solutions were filtered through a 0.45 µm membrane (Millipore, Billerica, MA, USA). Samples for HPLC analysis were then prepared.

### 3.4. Determination of Total Flavonoids Content

The method of determination of flavonoids content was a colorimetric assay. To rutin samples, with the same volume and different concentration were successively added 5% NaNO_2_ (0.3 mL, 6 min), 5% Al(NO_3_)_3_ (0.3 mL, 6 min), 4% NaOH (4.4 mL, 12 min). According to the optical density (OD) at 510 nm, calibration curves for rutin was drawn with the software Origin 7.5. This would give A, B and R^2^. Total flavonoids content was determined the same way as rutin. The formula used was as follows:

Total flavonoids content (%) = [(OD_1_ + OD_2_ + OD_3_)/3 − A]/B × 10/2 × volume/1000 × 100%

### 3.5. Antioxidant Activity

#### 3.5.1. DPPH Assay

The DPPH free radical scavenging activity of the extracts was measured according to our previous report [21]. Briefly, a solution of DPPH (0.1 mM) in methanol was prepared and 1 mL added to different concentrations of extract sample (1.0 mL). The mixture was shaken vigorously and incubated for 30 min in the dark. The absorbance value was measured at 517 nm. In the control, methanol was substituted for sample. The inhibitory ratio (%) was calculated by the following equation:

DPPH scavenging percentage (%) = (1 − A_sample517_/A_control517_) × 100

All the determinations were performed in triplicate and found to be reproducible within the experimental error.

#### 3.5.2. ABTS Assay

The ABTS assay of the extracts was performed according to our previous report [21]. ABTS and potassium persulfate were dissolved in ultrapure water to a final concentration of 7 mM and 2.45 mM, respectively. The mixture was allowed to remain in the dark for 12 h before use. Then, 500 μL extract samples of different concentrations were added to appropriately diluted ABTS solutions; the absorbance at 734 nm was read after 6 min. In the control, methanol was substituted for sample. The inhibitory ratio (%) was calculated by the following equation:

ABTS scavenging percentage (%) = (1 − A_sample734_/A_control734_) × 100

All the determinations were performed in triplicate and found to be reproducible within the experimental error.

#### 3.5.3. Reducing Power Assay

The reducing power of the extracts was quantified by the method described by Xiao *et al.* [34]. Extract samples of various concentrations (1 mL) were mixed with 2.5 mL phosphate buffer (PH = 6.0) and 2.5 mL of potassium ferricyanide (1%, *w*/*v*) at 50 °C for 20 min. 10% TCA was used to terminate the reaction. The mixture was centrifuged at 3000 rpm for 10 min, then 2.5 mL of supernatant, together with 2.5 mL of distilled water and 0.5 mL of 0.1% ferric chloride were mixed, and the absorbance was read at 700 nm. The mixture of 2.5 mL of supernatant and 3 mlL of distilled water was taken as blank. Every experiment was done in triplicate (*n* = 3) and found to be reproducible within the experimental error (RSD < 5.0%).

#### 3.5.4. Superoxide Anion (O^2ˉ^) Scavenging Activity

The measure of superoxide anion (O^2−^) scavenging activity was carried out as described previously by Xiao *et al.* [34]. Superoxide anion (O^2−^) was generated from 3.0 mL of sodium phosphate buffer (100 mM, PH = 7.4), which contained 1.0 mL of extract of various concentrations, 1.0 mL of NBT (150 μM) and 1.0 mL of NADH (468 μM). With the addition of 1.0 mL of PMS (60 μM), the reaction started and the mixture was incubated at 25 °C for 5 min. The absorbance at 560 nm was recorded. Capability to scavenging superoxide anion (O^2−^) was calculated using the formula:

Superoxide anion scavenging activity (%) = (1 − A_1_/A_0_) × 100

where A_0_ is the absorbance of the control sample, and A_1_ is the absorbance of samples. Each sample was tested three times (*n* = 3). The absorbance was found to be reproducible within the experimental error.

#### 3.5.5. FRAP Assay

The FRAP assay of the extracts was carried out according to our previous report [35]. The FRAP reagent contained 10 mM TPTZ in 40 mM HCl solution and 20 mM FeCl_3_ in 0.25 L acetate buffer (pH 3.6). It was freshly prepared and warmed to 37 °C. Briefly, 1.5 mL of FRAP reagent was added to 50 μL of extract of variable concentration. The absorbance at 593 nm was read after 4 min. The FRAP assay was expressed by using FeSO_4_ calibration curves. All the determinations were performed in triplicate and found to be reproducible within the experimental error.

### 3.6. AChE Inhibitory Activity

AChE inhibition activity of the flavonoid extracts was measured by the method adopted by Xiao *et al.* [36]. AChE was added to a mixture which contained 140 μL of sodium phosphate buffer (pH = 8.0), 20 μL of DTNB and 20 μL of tested extract and then incubated at 25 °C for 15 min. When this time is up, acetylthiochline (10 μL) was added to the mixture to activate the reaction. Finally, AChE inhibitory activity was evaluated by the percentage of AChE activity rate reduction from 100%. Each sample was tested three times (*n* = 3, RSD < 5.0%).

### 3.7. LC/DAD/ESI–MS Analysis

The LC-DAD-ESI/MS instrument consisted of an Agilent 1100 HPLC equipped with a diode array detector and an Agilent mass spectrometer (LC/MSD SL) (Agilent Technologies, Santa Clara, CA, USA). A Symmetry column (C18, 250 × 4.6 mm, 5 μm) (Waters, Milford, MA, USA) was used at a flow rate of 1.0 mL/min. The column oven temperature was set at 25 °C. The mobile phase consisted of 0.2% formic acid (**A**) and acetonitrile (**B**) with the following gradient program: 0–10 min, 5% B; 10–15 min, 5%–15% B; 15–25 min, 15% B; 25–35 min, 15%–25% B; 35–40 min, 25% B; 40–50 min, 25%–35% B; 50–60 min, 35% B; 60–70 min, 35%–50% B; 70–80 min, 50%–5% B. The flow-rate was kept at 0.30 mL/min. The DAD was set at 254 nm to provide real time chromatograms and the UV/Vis spectra from 190 to 650 nm were recorded for plant component identification. Mass spectra were simultaneously acquired using electro-spray ionization in the positive (PI) and negative ionization (NI) modes, at low (70 V) and high fragmentation voltages (250 V) for both ionization modes. For brevity, the high and low fragmentation voltages of the PI and NI modes will be identified as PI250, PI70, NI250, and NI70 in the text. The mass spectra were recorded for the range of *m*/*z* 100–1000, a drying gas temperature of 350 °C, a nebulizer pressure of 50 psi, and capillary voltages of 4000 V for PI and 3500 V for NI, were used. The LC system was directly connected with MSD without stream splitting [37].

## Figures and Tables

**Table 1:** Flavonoids identified tentatively in the archegoniophore and the gametophyte of *M. polymorpha* L*.*

Peak No.	T_R_ (min)	MW	*m*/*z*	UV_λmax/nm_	Identification	Archegoniophore	Gametophyte
1	28.25	594	595.1 [M + H]^+^ 593.2 [M − H]^−^	292 344	Kaempferol-3-*O*-rutinoside	none	exist
2	49.78	608	609.2 [M + H]^+^ 607.1 [M − H]^−^	268 300 336	Chrysoeriol 7-*O*-neohesperidoside	none	exist
3	52.19	432	433.1 [M + H]^+^ 431 [M − H]^−^	268 288 340	Apigenin-7-*O*-β-d-glucoside	none	exist
4	55.82	622	623.1 [M + H]^+^ 621.1 [M − H]^−^	258 330	Baicalein 6,7-di-*O-*β-d-glucopyranuronoside	none	exist
5	55.87	286	287 [M + H]^+^ 285 [M − H]^−^ 571 [2M − H]	254 348	Kaempferol	exist	none
6	58.19	446	447.1 [M + H]^+^ 445 [M − H]^−^	268 336	Apigenin-7-*O*-β-d-glucuronide	none	exist
7	63.03	270	271 [M + H]^+^ 269 [M − H]^−^	268 338	Apigenin	none	exist
8	64.5	300	301.1 [M + H]^+^ 299 [M − H]^−^	266 340	Chrysoeriol	exist	none
9	64.98	462	463 [M + H]^+^ 461 [M − H]^−^	254 348	Luteolin 3′-*O*-β-d-glucuronide	exist	none
10	68.50	638	639.1 [M + H]^+^ 637.1 [M − H]^−^	210 266 336	Tricin-7-*O*-rutinoside	exist	none
